# Gene expression in diapausing rotifer eggs in response to divergent environmental predictability regimes

**DOI:** 10.1038/s41598-020-77727-2

**Published:** 2020-12-07

**Authors:** Eva Tarazona, J. Ignacio Lucas-Lledó, María José Carmona, Eduardo M. García-Roger

**Affiliations:** Institut Cavanilles de Biodiversitat I Biologia Evolutiva, Universitat de València, Valencia, Spain

**Keywords:** Computational biology and bioinformatics, Ecology, Evolution

## Abstract

In unpredictable environments in which reliable cues for predicting environmental variation are lacking, a diversifying bet-hedging strategy for diapause exit is expected to evolve, whereby only a portion of diapausing forms will resume development at the first occurrence of suitable conditions. This study focused on diapause termination in the rotifer *Brachionus plicatilis* s.s., addressing the transcriptional profile of diapausing eggs from environments differing in the level of predictability and the relationship of such profiles with hatching patterns. RNA-Seq analyses revealed significant differences in gene expression between diapausing eggs produced in the laboratory under combinations of two contrasting selective regimes of environmental fluctuation (predictable vs unpredictable) and two different diapause conditions (passing or not passing through forced diapause). The results showed that the selective regime was more important than the diapause condition in driving differences in the transcriptome profile. Most of the differentially expressed genes were upregulated in the predictable regime and mostly associated with molecular functions involved in embryo morphological development and hatching readiness. This was in concordance with observations of earlier, higher, and more synchronous hatching in diapausing eggs produced under the predictable regime.

## Introduction

The variability that characterizes natural environments confronts organisms with the vital challenge of surviving during temporarily adverse periods. To cope with such adversity a widespread strategy among metazoans is diapause^1^. As a kind of dormancy, diapause permits escape in time through the temporal suspension of metabolic activity, arrested development and increased stress resistance^2,3^. In contrast to quiescence (another kind of dormancy controlled exogenously and implying an immediate response to a change in environmental conditions^4,5^), diapause depends on endogenous control for its initiation and termination^6^. Thus, diapausing organisms enter the passive state even under optimal environmental conditions that would otherwise promote normal metabolism and development, typically preceding the onset of adverse conditions^7^. In diapause, metabolic arrest remains for an internally scheduled period of time, the so-called refractory period^8^, even if suitable conditions resume. Only after the refractory period is completed do specific environmental cues disrupt diapause to restore active development and reproduction^9,10^. However, the decision to leave diapause remains a challenge if the cues anticipating future environmental conditions are unreliable and lead with some probability to zero-fitness events (death and reproductive failure in the case of annual organisms)^11^. To reduce the risk associated with such unpredictability organisms can employ a bet-hedging strategy for waking the diapausing forms whereby only a portion of these resume development at the first occurrence of cues for suitable conditions while the rest wait for later opportunities for reactivation^12–14^. The adequacy of diversifying bet-hedging strategies in leaving diapause (a single genotype producing a variety of phenotypes with different diapause durations^15^) to cope with unpredictable environments has received increasing support in the form of empirical studies^16–18^. Access to high-throughput next-generation sequencing (NGS) technologies can motivate studies pursuing better understanding of the molecular mechanisms behind the optimal duration of diapause.

Although it may be not realistic to speak of discrete steps in diapause, a convenient simplification is to divide it into the overlapping ecophysiological phases of initiation, maintenance and termination^19^. Initiation may be driven by differential gene expression leading to altered feeding regimes, changes in the synthesis and activity of cell proteins, and modification of behaviour and/or morphology. Subsequent to initiation, organisms enter the maintenance phase, which coincides with the greatest stress tolerance and the deepest dormancy, the intensity of each varying substantially between organisms^3,20^. While the molecular mechanisms of diapause initiation and maintenance have long attracted scientific attention and generated a considerable body of knowledge^2,21–25^, the mechanisms of diapause termination remain unclear, probably in part due to the intrinsic variability associated with bet-hedging strategies^26^.

In this contribution we focus on diapause termination in the monogonont rotifer *Brachionus plicatilis* sensu stricto, an important model organism for evolutionary and ecological studies^27,28^, in which bet hedging in diapause-related traits has been explored in some detail^17,18,29^. Monogonont rotifers are facultatively sexual aquatic microinvertebrates that produce diapausing forms as a by-product of sexual reproduction. These forms are encysted embryos called diapausing or resting eggs, so diapause in these rotifers is limited to the embryonic stage, in contrast to other organisms such as insects in which diapause can occur at any stage of their life cycle^30–32^. Once produced, diapausing eggs typically sink to the sediment of water bodies where they are able to persist under harsh environmental conditions such as drought, extreme temperatures, and salinity fluctuations^4,33^. Recently, some studies have developed genomic resources for *B. plicatilis*, contributing to the discovery of genes that are key for survival during diapause^23,25,34–36^. These genes are related to processes maintaining the stability and the integrity of cell compartments and macromolecules during this metabolically arrested stage of the rotifer life cycle (for review see^1^). The molecular mechanisms underlying diapause termination and subsequent hatching are in general unknown, although there is some evidence that a number of genes involved in embryonic development are expressed at this phase^35^. According to bet-hedging theory the optimal diapausing egg hatching fraction is related to the probability of facing favourable conditions when resuming development^29^. Therefore, diapausing eggs from environments differing in degree of predictability are expected to show different hatching phenotypes, translatable into terms of differential gene expression.

Our aim in this study is to provide insights into the transcriptional profile of diapausing eggs in response to divergent experimental regimes of environmental unpredictability and to relate this profile to observed hatching patterns. For this purpose, we used RNA-Seq to identify and analyse gene expression differences between diapausing eggs produced under combinations of (1) two contrasting selective regimes of environmental fluctuation (predictable vs. unpredictable) simulated in the laboratory, and (2) two different experimental diapause conditions (passing or not passing through a period of forced diapause in which hatching was inhibited). After a period of forced diapause, it is expected that a higher proportion of diapausing eggs will have exceeded the refractory period for obligate diapause and that they would reactivate immediately under proper hatching conditions. In parallel, subsamples of diapausing eggs produced under the abovementioned selective regimes were used to perform hatching experiments under the two diapause conditions, to relate hatching and gene expression patterns to each combination of predictability regime and diapause condition. Taking into account the divergent pattern expected in the hatching fraction between selective regimes (i.e., higher hatching fractions in a predictable regime compared to unpredictable^18^), we hypothesize that the genes involved in diapause termination will be more highly expressed under the predictable regime. In contrast, low hatching fractions observed in diapausing eggs produced under the unpredictable regime suggest that many of these eggs remain under metabolic and development arrest. To consider the mechanisms translating environmental unpredictability into a lower hatching fraction, we formally test the hypothesis that an overall higher variation in gene expression mediates higher uncertainty in the time of hatching.

## Results

### Diapausing egg hatching experiment

**Figure 1:**
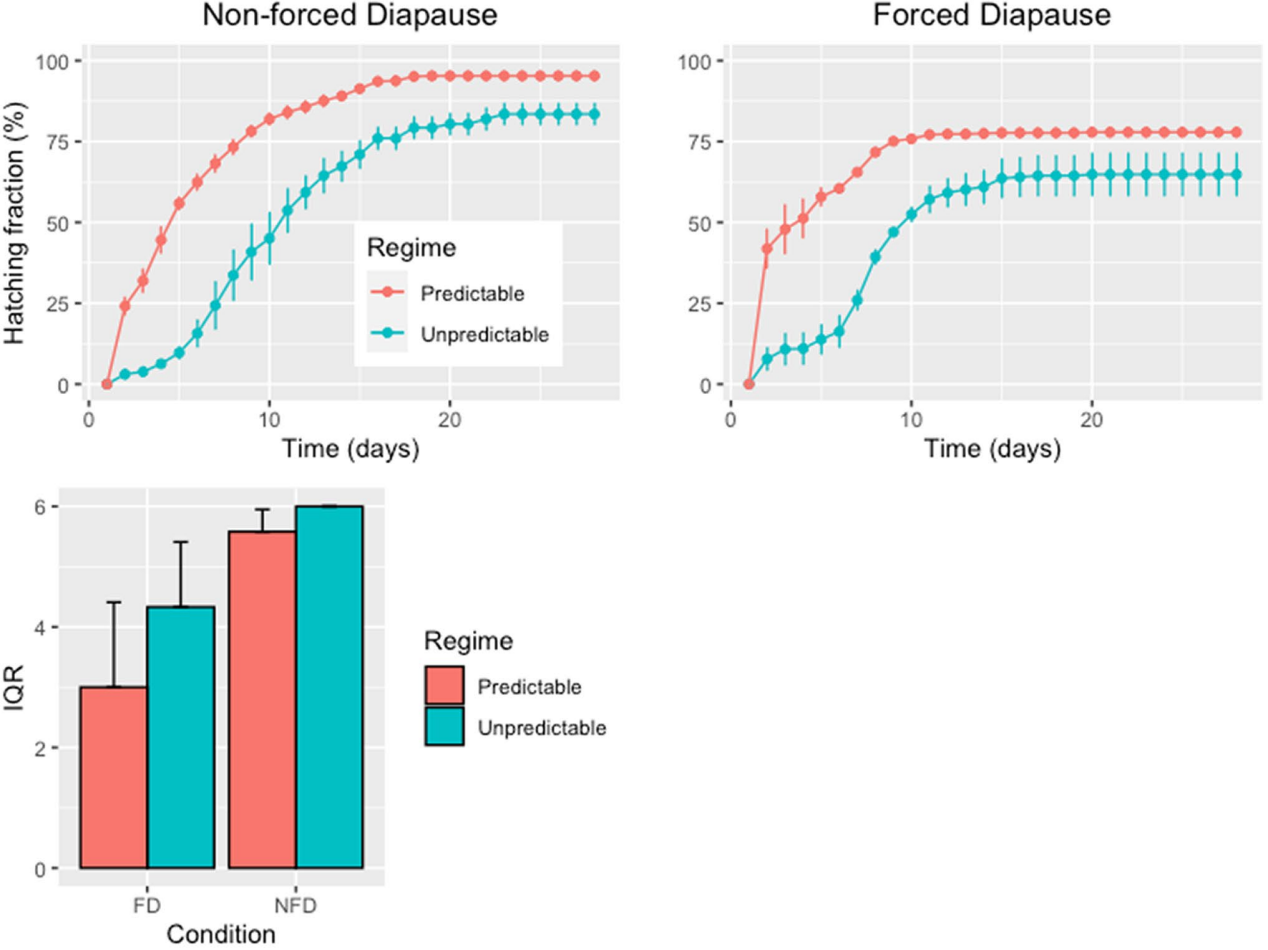
Cumulative percent hatching of *Brachionus plicatilis* s.s. diapausing eggs obtained under two laboratory selective regimes (predictable and unpredictable) after 28 days of incubation under two diapause conditions: (**A**) non-forced diapause and (**B**) forced diapause. (**C**) Asynchrony of diapausing egg hatching in *Brachionus plicatilis* s.s. as estimated by the interquartile range (IQR) in each combination of selective regime (predictable vs unpredictable) and diapause condition (forced diapause [FD] vs non-forced diapause [NFD]). Bars indicate ± 1 SE.

Both predictability regime and diapause condition affected diapausing egg hatching fraction. The effects of both factors and their interaction were significant (Table 1). Hatching fractions were significantly lower under the unpredictable regime for both diapause conditions (Non Forced Diapause (NFD) and Forced Diapause (FD)) (Fig. 1A,B). Timing of hatching was also significantly affected by selective regime (survival log-rank test; *χ*^2^ = 236, *p-*value < 0.001) and diapause condition (*χ*^*2*^ = 28.1, *p-*value < 0.001). We observed a delayed hatching pattern in populations from the unpredictable regime, with a median hatching time (H_50_) averaging 9.66 ± 1.20 days and 8.00 ± 0.57 days under NFD and FD conditions, respectively. Under the predictable regime, H_50_ values averaged 4.66 ± 0.33 days and 2.66 ± 0.66 days for NFD and FD conditions, respectively. No matter the selective regime, FD condition resulted in significantly lower values for the interquartile range of hatching time (IQR, here taken as a measure of hatching synchronicity) compared with the NFD condition (Table 1; Fig. 1C). Whatever the experimental condition, IQR was always higher in the unpredictable regime than in the predictable one (Fig. 1C).

**Table 1:** Summary of the generalized linear mixed-effect model (GLMM) on diapausing egg hatching fraction, and the linear mixed-effect model (LMM) for the interquartile range (IQR) of hatching times.

Effect	Hatching fraction		IQR	
	*χ2*	*p*-value	*χ2*	*p*-value
Regime	28.047	< 0.001	1.137	0.287
Population (Regime)	1.398	0.284	–	–
Condition	97.367	< 0.001	10.616	0.001
Regime × Condition	6.997	0.008	0.494	0.482

Note that the significance of the “population” effect for the diapausing egg fraction was tested using a likelihood ratio test (LRT; see “Material and methods”).

### Mapping and alignment of RNA-Seq reads to the *B. plicatilis* s.s. genome

High-throughput sequencing generated 24.8 G of raw reads. The number of filtered reads and percentage of these that mapped to the reference *B. plicatilis* s.s. genome for each population are shown in Table 2. An average of 36,896 ± 388 expressed genes were identified for each combination of predictability regime and diapause condition (U-NFD, U-FD, P-NFD, P-FD). A quality control plot of RPKM density distribution (the squared coefficient of variation (CV^2^) vs log_10_FPKM) for each combination of predictability regime and diapause condition is shown in Supplementary Figure S1. Overall, the variability in gene expression levels in diapausing eggs produced under the unpredictable regime was always higher than in those produced under the predictable regime, whatever the diapause condition tested.

**Table 2:** Statistics for the filtering and mapping of RNA-Seq reads corresponding to combinations of selective regimes and diapause conditions.

Regime/condition	Population	Reads	Reads mapped
U-NFD	1	58,364,853	46,103,494 (79.0%)
	2	55,711,941	49,369,286 (88.6%)
	4	59,268,714	51,793,879 (87.4%)
	3	55,778,100	48,926,714 (87.7%)
P-NFD	5	39,381,051	30,637,627 (77.8%)
	6	55,388,301	48,681,614 (87.7%)
	1	29,222,289	25,433,737 (87.0%)
U-FD	2	38,563,452	34,283,803 (88.9%)
	4	37,364,770	32,119,977 (86.0%)
	3	44,386,767	38,807,814 (87.4%)
P-FD	5	36,745,427	31,601,100 (86.0%)
	6	42,730,345	37,107,511 (86.8%)

*P* predictable regime, *U* unpredictable regime, *NFD* non-forced diapause, *FD* forced diapause.

### Variation partitioning analysis of gene expression

Differentially expressed genes (DEGs) associated with the “regime” and “diapause condition” factors, and their interaction, were assessed by (1) the log_2_-fold change (log_2_-FC) values between the levels of each factor and their associated *p*-values, and (2) by the proportion of expression variance explained by each factor (see “Material and methods”). Our analysis also allowed testing the random effect of population within each selective regime. A total of 18,598 genes were scored and assigned a variance fraction and a differential expression *p*-value.

**Figure 2:**
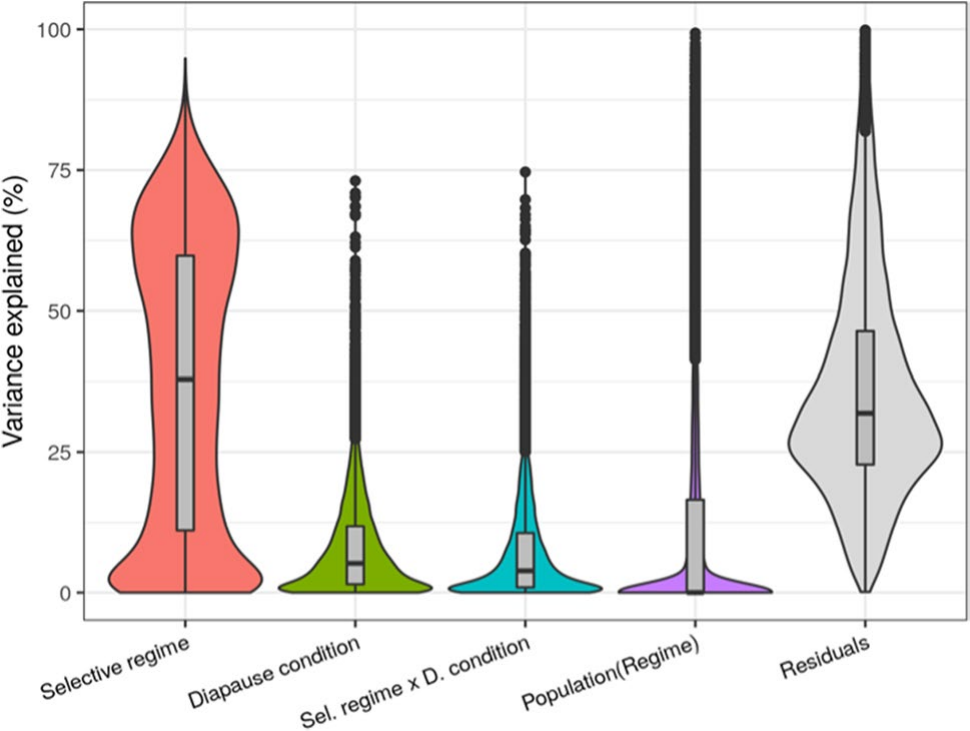
Violin plots of the variance in gene expression in diapausing eggs of *Brachionus plicatilis* s.s. explained by different factors (%).

The relative amounts of variance in gene expression explained by the predictors of our model are summarized in Fig. 2. Overall, we observed that the “regime” factor accounted for the highest proportion of the variation in gene expression in *B. plicatilis* s.s. diapausing eggs, with a median percentage close to 40%. In contrast, the “diapause condition” factor and its interaction with “regime” explained approximately 10% of variance in gene expression. The random effect of population within regime was negligible (Fig. 2).

Consistent with analysis of variance partitioning, post hoc corrections of the associated *p*-values of differential gene expression showed that 76% of the whole set of expressed genes retrieved in our analysis were not differentially expressed between selective regimes. No gene was found to be differentially expressed between diapause conditions after *p*-value correction for multiple comparisons. Since both approaches strongly suggest that diapause condition was not as important in driving differences in the transcriptional profile of diapausing eggs than the selective regime, hereafter we focused only on the latter factor.

### Variation in gene expression levels among replicates

The estimated biological variation in gene expression levels among replicates follows a very similar trend along the range of average expression values in the two regimes, with much higher variance among genes expressed at very low levels (Supplementary Figure S2). Only among those low-expression genes, expression level is more variable among replicates in the unpredictable compared with the predictable regime. When comparing whole expression profiles between samples, a test of homogeneity of dispersion based on permutations fails to detect any significant difference between the two regimes (*p*-value = 0.883; Supplementary Figure S3).

### Gene function assignment and GO enrichment analysis of DEGs

To find specific functions related to diapause maintenance or to embryonic development (diapause termination) we inspected GO terms significantly enriched with genes differentially expressed between predictability regimes following the ordination provided by the variation partitioning analysis. Notably, both approaches used for gene ordination yielded similar results (Supplementary Figure S4). A total of 9691 DEGs had GO annotations. In our inspection we included only the most specific GO terms assignable to a gene and skipped those that were too vague or general. Supplementary Tables S1, S2 and S3 show the GO specific terms that were significantly associated with the “regime” factor according to all three algorithms used in the enrichment analysis for biological processes (GO:0008150), molecular functions (GO:0003674), and cellular components (GO:0005575).

**Figure 3:**
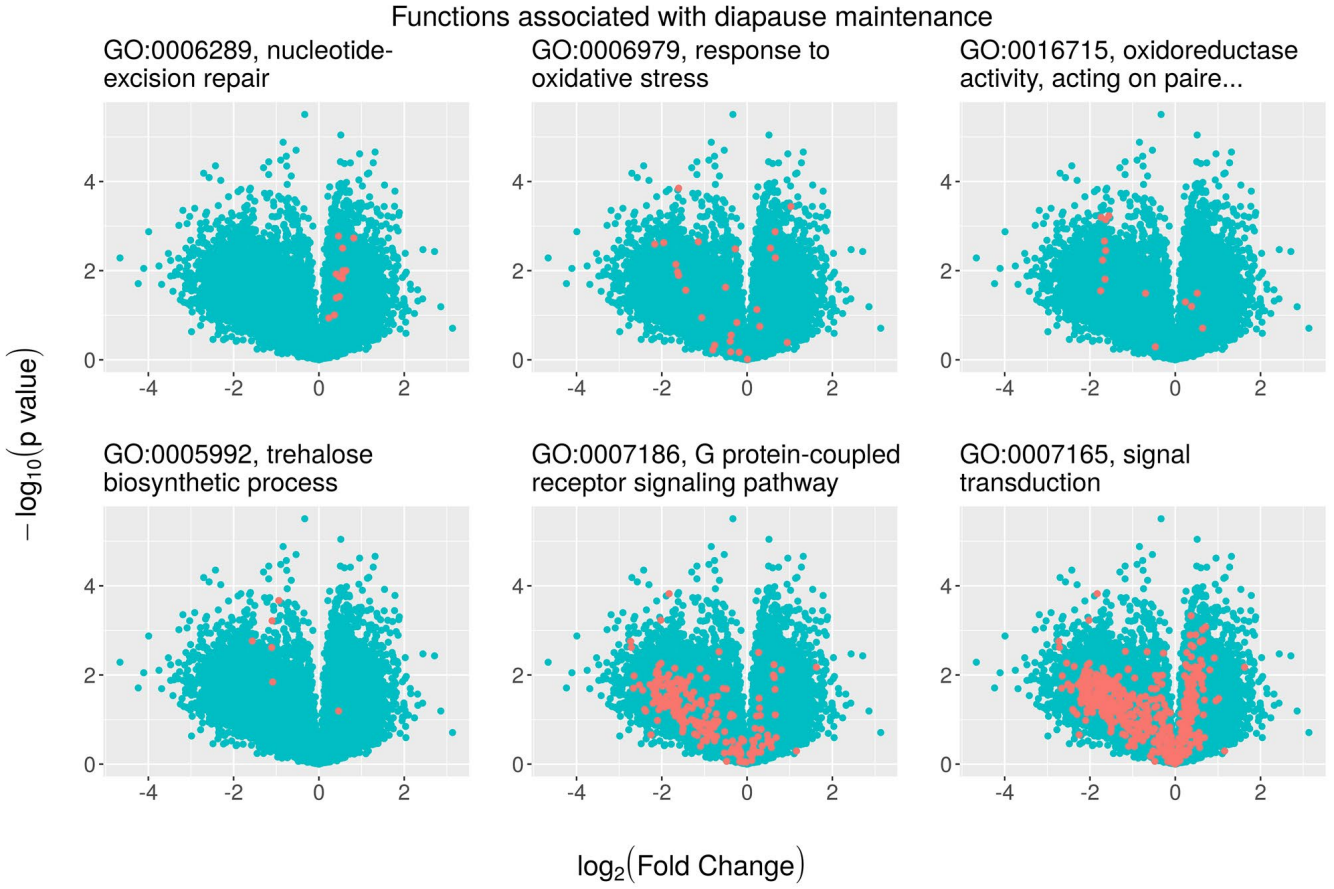
Volcano plots (statistical significance vs. fold change, FC) showing differential gene expression associated with selective regimes for different gene ontology (GO) specific terms related to diapause maintenance (see text for description). Each point is a separate gene. The red dots highlight genes included in each particular GO pathway (see headings) over the entire set of differentially expressed genes among regimes. In all plots, positive values of log_2_-FC indicate upregulation in the unpredictable regime. If log_2_(FC) is negative, gene expression is higher in the predictable regime.

**Figure 4:**
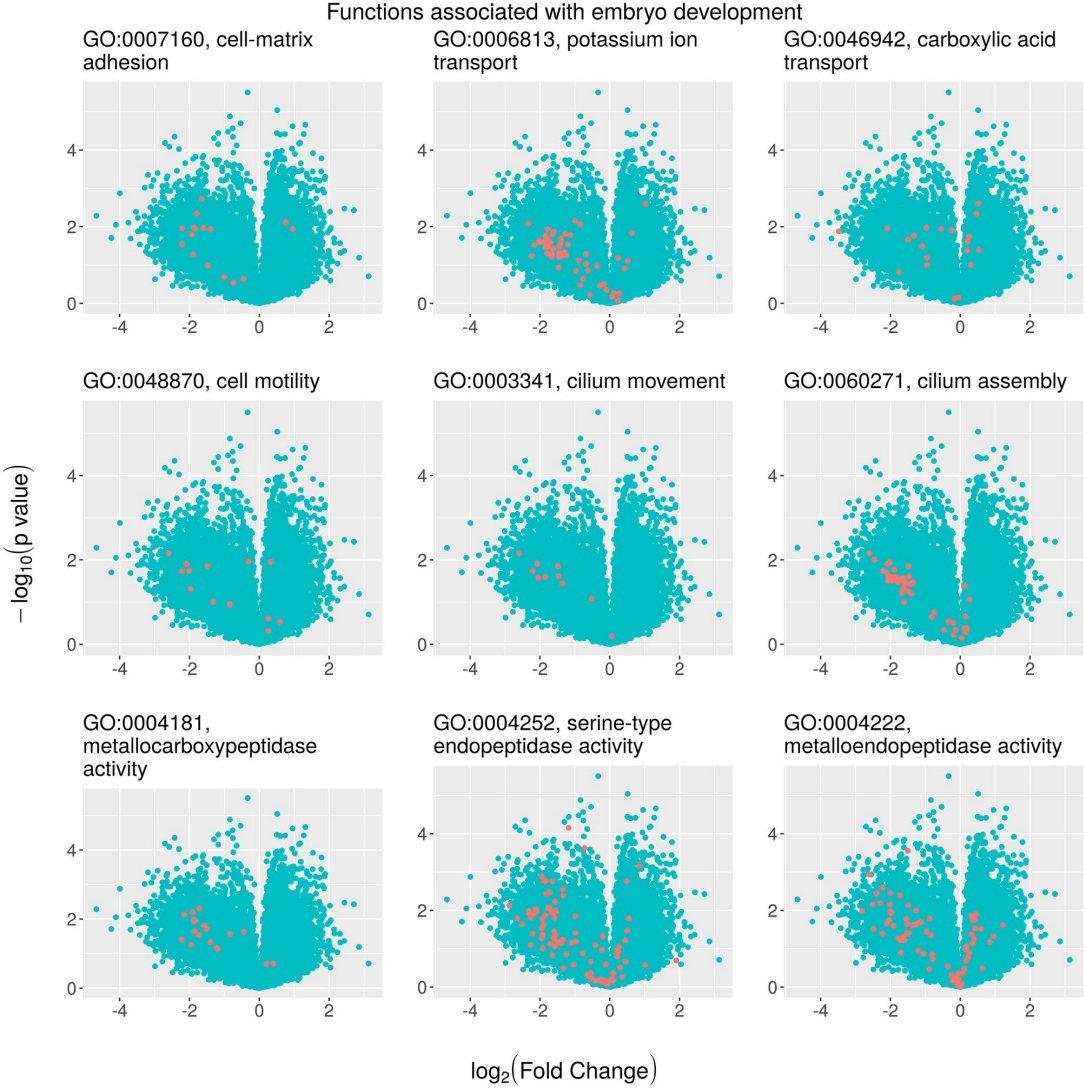
Volcano plots (statistical significance vs fold change, FC) showing differential gene expression associated with selective regime for different gene ontology (GO) specific terms related to embryo development (diapause termination). Each point is a separate gene. The red dots highlight genes included in each particular GO pathway (see headings) over the entire set of differentially expressed genes among regimes. In all plots, positive values of log_2_-FC indicate upregulation in the unpredictable regime. If log_2_(FC) is negative, gene expression is higher in the predictable regime.

First, we made a tentative assignment of GO terms to diapause phases on the basis of previous knowledge. Proper references and rationales for GO term assignment to different phases of diapause are shown in Table 3. Next, we used volcano plots for GO specific terms associated with the “regime” factor to better demonstrate the role of DEGs in diapause maintenance (Fig. 3) and termination (Fig. 4). The volcano plots indicate the size of the biological effect (fold change) versus the statistical significance of the result (*p*-value) in association with the unpredictable regime. Positive log_2_-FC values indicate upregulated genes in the unpredictable regime with respect to the predictable one, thus negative values are indicative of under-expression. These plots were intended to help identify subsets of genes involved in different functions within the entire set of DEGs, and to focus further research on the most promising.

**Table 3:** Gene ontology (GO) term assignment and rationale to the different phases of diapause.

Diapause phase	GO term	Rationale
Diapause maintenance	Nucleotide-excision repair (4/13)	Known as a repair mechanism in desiccation tolerant stages^37^. Desiccation/rehydration cycles and prolonged periods in the dry state are associated with remarkable levels of stress to the embryo genome which can result in mutagenesis of the genetic material, inhibition of transcription and replication and delayed growth and development
	Response to oxidative stress (10/26)	Oxidative stress leads to the accumulation of toxic components called reactive oxygen species (ROS). Therefore, production of antioxidant enzymes is critical for stress resistance during diapause^38^ and has been shown in a wide range of organisms such as insects^39^, nematodes^40^ and rotifers^34^. The major ROS detoxifying enzymes described in rotifers include peroxidases, thioredoxins, catalases and glutathione-S-transferases^34,36^
	Oxidoreductase activity (63/147)	A number of proteins with oxidoreductase activity have been described as involved in diapause with a role in detoxification processes and degradation of xenobiotics^36^. For example, the cytochrome P450 monooxygenase gene has been shown to be overexpressed in insects during diapause^3,5^. In the case of monogonont rotifers this gene is also related to lipid metabolism (steroidogenesis^41^) with a potential role in the accumulation of energy reserves for diapause^42^
	Trehalose biosynthetic process (4/6)	Trehalose is related to dormancy in many prokaryotes and eukaryotes, and is known to be synthesized under dehydration conditions to stabilize other proteins and membranes during drying^41,43,44^. Trehalose acts as a water replacement molecule and vitrifying agent serving to stabilize cell membranes^45^. Trehalose is known to accumulate in diapausing cysts of *Artemia*^45,46^ and in diapausing rotifer eggs when they enter anhydrobiosis^47^. Transcripts encoding for trehalose-6-phosphate synthase and for trehalose phosphate synthase enzymes have been detected in diapausing rotifer eggs, suggesting its biosynthesis in rotifers^23,25,34^
	G protein-coupled receptor signalling pathway (17/206)	G protein-coupled receptors (GPCR) regulate signal transduction pathways and play diverse and pivotal roles in the physiology of insects, especially in resistance to adverse environmental conditions^48^. Interestingly, a GPCR receptor in the silkworm, *B. mori*, has been identified as a specific cell surface receptor for the diapause hormone (DH)^49^. In the nematode, *C. elegans*, two transcripts for GPCRs have been identified as among the most abundant in the dauer stage^50^
	Signal transduction (45/544)	Signal transduction pathways (insulin signalling) are known to have a role in the entrance into and maintenance of the dauer state in nematodes^51^, and diapause in insects^52^ and killifish^53^
Embryo development	Cell–matrix adhesion (3/16)	The interactions of cells with the extracellular matrix play crucial roles during morphogenesis in developing embryos. Interestingly, Clark et al.^36^ have reported the upregulation of genes involved in cell proliferation and adhesions in the transcription profile of non-diapause embryos in their morphological development into neonate rotifers
	Potassium ion transport (4/56)	K^+^ is known to have a role in cell growth through protein synthesis. There is evidence of K^+^-mediated stimulation of aminoacyl-tRNA (aa-tRNA) synthetase activity in bacteria^54^ and insects^55^, with an effect on diapause termination in the latter. Rozema et al.^25^ have shown high levels of aa-tRNA synthetases in diapausing compared to asexual eggs in *Brachionus*
	Ion transmembrane transport (16/147)	Free ions in the cytosol restart the cell machinery by reactivating cellular enzymes^56^. Dumont et al.^57^ showed that cyst hatching in some anostraceans increased after the addition of mobile ion carriers. There is also evidence that increased pH induces diapause termination in *Artemia salina*^26^. Interestingly, transmembrane proton transport, and H^+^-translocating pyrophosphatase activity has been reported in *Brachionus* diapausing embryos after 30 min of light stimulation for hatching^58^
	Nucleoside transmembrane transport (1/7)	Nucleosides, precursors of the nucleotides, would provide the material needed to generate new DNA and RNA for cell proliferation in resumed development. In addition, nucleosides (and nucleotides) are key determinants of energy metabolism, ligands for purinergic receptors, and transducers of endocrine signals
	Carboxylic acid transport (2/20)	Metabolic depression in diapausing rotifer embryos may be attributed to a putative downregulation of the tricarboxylic acid (TCA) cycle and pyruvate metabolism^23,25^. Consequently, upregulation of this pathway is expected in diapause termination and development reactivation
	Cell motility (1/14)	Cell motility has been related to the resumption of embryonic development after diapause, and changes in the expression of genes encoding for cytoskeletal components (actin and tubulin) have been reported in bdelloid rotifers^59^ and zebrafish^60,61^. Clark et al.^36^ have reported the expression of an actin-binding protein with a role in the regulation of cytoskeletal organization in diapausing *Brachionus* eggs
	Cilium assembly (2/46) and movement (1/10)	Cilium assembly and movement are known to be regulated by the cytoskeleton. Interestingly, one way to recognize the viability of rotifer embryos after diapause is through microscopic observation of the coronal cilia beat (personal observation)
	Proteasome mediated ubiquitin dependent protein catabolic process (4/45)	This pathway includes chemical reactions resulting in the breakdown of proteins by hydrolysis of peptide bonds. Whether this is a way to obtain energy for a resource-intensive process such as hatching is yet to be resolved. Genes associated with protease activity are rapidly upregulated in diapausing *Brachionus* eggs after 30 min of light stimulation for hatching^58^
	Motor activity (11/131)	Likely related to resumption of morphological development in rotifers and diapausing egg hatching, the function is defined as the generation of force resulting in movement along a microfilament or microtubule coupled to the hydrolysis of a nucleoside triphosphate
	Peptidase activities (59/399)	Protein catabolism may constitute a source of amino acids for development after diapause. Several peptidases have been reported to be overexpressed in diapausing *Brachionus* eggs shortly (30 min-4 h) after receiving a light stimulus for hatching^58^

Numbers in parentheses denote the number of significant differentially expressed genes (*p*-value < 0.01) out of the total number of genes annotated with that term.

A rough estimate of the level of activity of a molecular pathway is the number of genes differentially expressed between the selective regimes. By this criterion, two pathways stand out among the a priori functions assigned to the maintenance of diapause (Fig. 3): G protein-coupled receptor signalling (GPCR) and signal transduction. There are 206 genes differentially expressed in the GPCR pathway (55 with an adjusted *p*-value lower or equal to 0.1) and 544 genes involved in signal transduction. Of course, not all molecular pathways are equally complex, and a better approach involves comparing the fraction of genes differentially expressed in a given regime. Thus, for instance, the nucleotide-excision repair term is among the most significantly enriched terms with 13 genes annotated. Notably, all 13 were overexpressed in the diapausing eggs from populations evolved in the unpredictable regime. For other functions with a putative role during diapause, including those involved in protection and isolation from external stimuli, the change in expression was not in the same direction for all genes that were regulated by the selective regime; however, we observed that higher numbers of genes were in general expressed in diapausing eggs from populations evolved under the predictable regime.

Functions associated with embryo development included pathways related to (1) cellular association with the extracellular matrix and cell motility, (2) cilium assembly and motility, (3) ion transport, and (4) protein catabolism. We observed that most genes in the pathways were underexpressed in the unpredictable regime (Fig. 4). Overall, for the set of functions depicted in Fig. 4, the proportion of genes that showed increased expression in the predictable regime averaged 77.8 ± 3.9%.

## Discussion

This study focuses on a highly relevant stage of the life cycle of *B. plicatilis* s.s*.*, the diapausing egg stage, and explores patterns of diapause maintenance and termination. We combined hatching experiments and transcriptomics to evaluate diapausing eggs produced by populations evolving under two divergent regimes of environmental predictability and subjected to conditions either promoting or blocking hatching. Different hatching patterns were observed between selective regimes and diapause conditions and more than 3000 genes with different expression profiles between the studied comparisons were identified. To the best of our knowledge, this is the first study that uses RNA-Seq and a reference genome to relate hatching patterns to transcriptional profiles in diapausing eggs subjected to predictable and unpredictable laboratory environments.

Intermediate hatching fractions and variable diapause duration have been proposed as adaptations to environmental unpredictability in rotifers^11,18,29,33^. Diapausing eggs from populations subjected to a predictable regime showed higher, earlier and more synchronous hatching than those produced by populations under an unpredictable one. Interestingly, these traits have been observed to evolve quickly under the divergent predictability regimes assayed in our experimental evolution approach^18^. Consistent with the initial hypothesis, the timing of diapausing egg hatching was earlier and more synchronous after a forced period of diapause. A striking result was that hatching fractions were slightly lower in FD than under NFD conditions. This may be due to an increased diapausing egg mortality due to the forced diapause period^62,63^, despite the apparent health of all eggs in our assays, a trait that has been shown to correlate with their ability to hatch^64^.

Our results suggest that selective regime is more important in driving differences in the transcriptome profiles of diapausing eggs than the conditions of diapause. Interestingly, while the numbers of up- and downregulated DEGs are similar in either regime, the absolute fold change is on average twice as great in DEGs upregulated in the predictable regime. It is likely that most DEGs overexpressed in the predictable regime are related to the reactivation of diapausing eggs and hatching readiness. This would be in concordance with the results from the hatching experiment in which diapausing eggs produced under the predictable regime showed earlier and higher rates of hatching. Interestingly, after 30 h of exposure to hatching induction conditions embryo movement was observed in most of the diapausing eggs from the predictable regime (personal observation). The incidence of embryonic movement was lower in the diapausing eggs from the unpredictable regime. Correspondingly most genes associated with molecular functions related to morphological development of the embryo (cell–matrix adhesion, cell motility, cilium movement and cilium assembly) and cytoskeleton (motor activity) were upregulated in diapausing eggs obtained from populations subjected to the predictable regime under both diapause conditions.

We also found evidence of upregulation of protein catabolism-related genes (peptidase activity including metallocarboxypeptidases, metalloendopeptidases, serine-type endopeptidases and calcium-dependent cysteine-type endopeptidases) in diapausing eggs from populations subjected to the predictable regime. Protein catabolism may serve as a source of amino acids for development after diapause. These results have points in common with previous studies that explored the expressions of transcripts involved in protein turnover and synthesis of ATP and cytoskeletal elements^36^. Interestingly, research on copepods has also shown that proteins are increasingly utilized as an energy source by the end of diapause when neutral lipid storage becomes depleted^65,66^. It is known that the diapausing eggs of rotifers store fat reserves^42^ and it is likely that harvesting of lipids for the embryo starts at the time of diapausing egg production (diapause initiation); however, no molecular function associated with lipid metabolism had annotated DEGs that related to the predictability regime in our study. This could be because their expression is highly conserved in diapausing eggs independent of environmental predictability regime and conditions during diapause. Indeed, genes related to general lipid metabolism have been suggested to exert an ambivalent role in rotifer diapause^35^. Therefore, during diapause organisms may preferentially catabolize neutral lipids while conserving and synthesizing polar ones, which have an essential role in maintaining cell membrane structure and integrity. Interestingly, we found transcripts for the *fatty acid synthase* gene complex, known to be involved in lipid accumulation and stress tolerance during diapause in beetles^67^. A hypothesis to be tested is whether the usage of neutral lipids can be arrested during diapause and resumed exactly at the time of reactivation by using the stored lipids that have not been depleted during diapause. This makes sense in rotifers since large numbers of droplets with neutral lipids have been found in females that hatch from diapausing eggs^68^. This finding suggests a role for lipids in rotifer development after hatching and in the improvement of their ability to colonize new environments^42,69,70^.

We found a less probable, delayed, and less synchronous hatching pattern in diapausing eggs under the unpredictable regime than under the predictable regime under both diapause conditions. This hatching pattern is consistent with the general downregulation observed in the diapausing eggs from populations subjected to the unpredictable regime, possibly associated with developmental arrest during diapause^32,69,71^. The maintenance of diapause in a larger fraction of eggs or for a longer time under the unpredictable regime entails a higher uncertainty concerning the behaviour of the eggs, mirroring the uncertainty of the environment. We tested whether this more unpredictable behaviour in the diapausing eggs translated into more variable gene expression profiles among samples. Only genes expressed at very low level were observed to have more variable expression among samples from the unpredictable regime. The overall biological variation in gene expression levels among samples was of a similar magnitude in both regimes.

In general, it is expected that diapausing eggs that have not completed their mandatory diapause period may actively maintain the molecular pathways involved in the processes of repair, protection, and isolation from external stimuli^5,43,72^. Consequently, our specific prediction was that genes belonging to these pathways would be overexpressed in diapausing eggs from populations evolved under the unpredictable regime. Our results partially agreed with this prediction in the case of the expression of nucleotide-excision repair genes to reverse genome damage incurred during diapause. Monogonont rotifers are not unique in exhibiting this ability. Other metazoans that exhibit enhanced DNA repair capacity include tardigrades^73^ and bdelloid rotifers^74^. It has been hypothesized that such ability is a consequence of evolutionary adaptation to survive desiccation and exposure to high-energy radiation in ephemerally aquatic habitats^74^.

Functions providing protective mechanisms to the diapausing eggs in rotifers may have also evolved to withstand the physical effects of desiccation^23,34,36^. Since experimental populations of both regimes underwent desiccation-rehydration cycles, it is not surprising that we detected gene expression for some of these functions in their diapausing eggs. Consistent with previous reports on diapausing rotifer eggs, the GO terms associated with protective functions we retrieved in our analysis referred to pathways for trehalose biosynthesis (see also^23,34^), response to oxidative stress (thioredoxins*,* peroxidases, and glutathione-S-transferases^35,36^) and oxidoreductase activity (the *cytochrome P450* gene^1^). However, our results show that most of the genes involved in the three abovementioned pathways were more overexpressed in diapausing eggs from the predictable regime compared with those from the unpredictable one, which remains unclear. The fact that a few genes within these pathways were still overexpressed in the unpredictable regime suggests that diapausing eggs from populations evolved under different selective regimes may be using different sets of genes to protect themselves against desiccation or oxidative stress. This observation may be related to the probability that diapausing eggs from one regime or the other are in a different ecophysiological phase of diapause (maintenance or termination). Naturally, both developing embryos and rotifer hatchlings must have mechanisms to protect themselves against oxidative stress^75,76^. It is also conceivable that some genes are annotated as involved in a function that they regulate negatively, a hypothesis deserving further research.

While in diapause, rotifer embryos are less sensitive to external stimuli. Therefore, we expected that the molecular pathways responsible for environmental isolation would be more expressed in diapausing eggs that have not completed the refractory period compared with those that have resumed development. In relation to predictability regime, we hypothesized that a higher proportion of diapausing eggs from populations evolved under the unpredictable regime have not passed this period compared with eggs from populations evolved under the predictable regime. Contrary to our hypothesis, most of the genes in the signal transduction and GPCR signalling pathways were underexpressed in the unpredictable regime. Let us here recall the likely ambivalent role of some gene families in relation to environmental information processing. As mentioned above, rotifer diapausing eggs need a light stimulus to re-initiate development. In the absence of light, prostaglandins have been shown to cause the same effect^77^. Interestingly, some photoreceptors, such as the opsins, belong to the family of GPCRs, and prostaglandins are known to bind with a GPCR receptor.

In conclusion, our comparative transcriptome analysis, supported by hatching data, provided mechanistic insight into the adaptation of rotifer diapause to habitat unpredictability. Overall, we found evidence that molecular pathways related to diapause maintenance and termination were differentially expressed across rotifer diapausing eggs produced under two laboratory environments: predictable vs. unpredictable. Molecular functions related to embryo development reactivation were in general upregulated in diapausing eggs from predictable environments, consistent with the higher hatching fraction of eggs produced under this selective regime. However, some of the genes related to diapause maintenance were also upregulated in the predictable regime under both diapause conditions assayed. It is possible that expression of some molecular pathways is highly conserved in diapausing eggs independent of the selective regime and conditions during diapause but also that different sets of genes within a pathway are expressed at different phases during diapause^2^. Our study also extends knowledge of the complex molecular and cellular events that take place during diapause. Some of the genes identified here are well known in other anhydrobiotic organisms and resistance forms, but several are new and should be further investigated to determine their involvement in desiccation and stress tolerance. In this sense gene assignment of function to either diapause maintenance or embryonic development processes, although based on previous literature, is still tentative and further research is needed. In fact, several studies have shown that gene families traditionally associated with diapause maintenance, such as some glutathione-S-transferases, aquaporins, ferritins, or trehalose metabolism genes^34^, are highly expressed in diapausing eggs after exposure to 30 h of light^35^, so these genes may also be involved in embryonic development reactivation. Thus, we call for further research to achieve a better understanding of the present results and to further elucidate the genes related to diapause in unpredictable environments. Given future increases in environmental variability due to climate change^78^, understanding the molecular mechanisms underlying differential hatching patterns such as those found in the present and previous studies is essential to understand how organisms cope with environmental unpredictability.

## Material and methods

### Experimental design and diapausing egg collection

**Figure 5:**
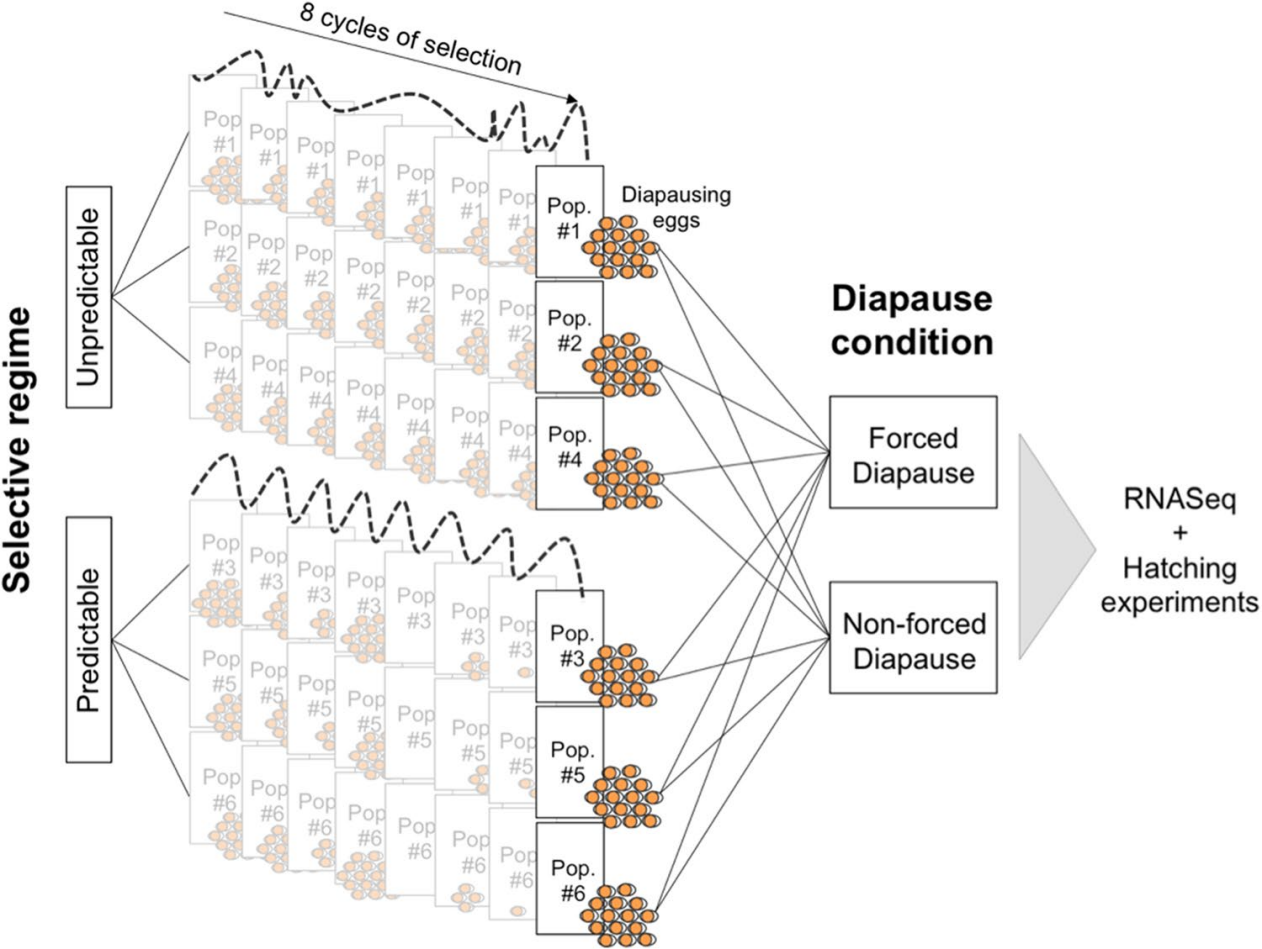
Split-plot experimental design followed in this study. “Regime” (two levels: predictable [P] vs unpredictable [U]) acts as the “whole-plot” factor. “Diapause condition” (with two levels: forced diapause [FD] vs. non-forced diapause [NFD]) acts as the split factor. Note that populations are nested within each regime and are subjected to 8 cycles of selection under either a predictable or unpredictable experimental hydroregime. Diapausing egg production is dependent on the length of each hydroperiod cycle. After the 8th cycle, diapausing eggs produced by each population were randomly separated into two samples, each subjected to either FD or NFD conditions.

Diapausing eggs were produced by laboratory populations of *B. plicatilis* s.s. cultured in an experimental evolution design that tested the effects of hydroperiod length unpredictability on diapause-related traits. Briefly, six genetically diverse laboratory populations were generated by placing together three ovigerous asexual females from each of 30 clonal lines of *B. plicatilis s.s.* from nine natural populations (a total of 270 clones). Thus, the initial genetic composition of each of the six experimental populations was the same. Clonal lines were founded from the hatchlings of diapausing eggs isolated from the sediments of nine natural ponds in Spain covering a natural range of habitat predictability (see full details in^18^). Because *B. plicatilis* s.s. belongs to a cryptic species complex, clones were identified as belonging to *B. plicatilis* s.s*.* by genetic analysis of COI based on PCR–RFLP (see^17^). The six laboratory populations were subjected to two contrasting time-fluctuating laboratory environments, predictable (P) vs unpredictable (U), for eight growth cycles of selection (Fig. 5). These growth cycles were interrupted by periods of habitat unsuitability (dry periods) during which diapausing eggs were collected and then after a fixed period of obligate diapause (28 days) used to re-form each experimental laboratory population. Three laboratory populations were randomly assigned to the predictable selective regime, characterized by growing seasons (hydroperiods) of constant length (28 days). The other three laboratory populations were assigned to the unpredictable regime, characterized by growing seasons whose length varied randomly (between 4 and 53 days with an average of 28 days).

At the end of the last cycle of selection (the eighth growing cycle), diapausing eggs were collected, cleaned and counted. Diapausing eggs obtained from each laboratory population were randomly divided into two groups to be subjected to two different diapause conditions: (1) “non-forced diapause” (NFD) and (2) “forced diapause” (FD) (Fig. 5). For the first condition, a total of 5200 newly produced diapausing eggs per laboratory population were placed in Petri dishes and immediately induced to hatch under standard hatching conditions (constant light, 6 g L^−1^ salinity and 25 °C^64^) for 30–36 h. This period of time is considered to be enough to reactivate diapausing embryo development but not to complete hatching (see^35^; personal observation: no hatchlings were observed when handling the diapausing eggs). Thus, diapausing eggs were exposed to hatching conditions and not forced to remain in diapause (NFD). For the second condition a similar 5200 diapausing eggs per laboratory population were air-dried in darkness at 25 °C and then stored in darkness at 4 °C for 28 days. These conditions typically inhibit hatching^42,79^ so diapausing eggs were forced to remain in diapause (FD) during this period of time. After this period of forced diapause, diapausing eggs were placed in Petri dishes and induced to hatch under standard hatching conditions, as for the NFD condition, for 30–36 h. As depicted in Fig. 5 the experiment followed a split-plot design^80^ in which the “predictability regime” was the whole-plot factor, the experimental populations were the subplots nested within the whole plots, and “diapause treatment” was the so-called split-plot factor. A total of 12 diapausing egg samples resulting from 2 selective regimes (P vs U) × 3 laboratory populations within regime × 2 diapause conditions (FD vs NFD) were obtained to be further subjected to both hatching and gene expression analyses.

### Hatching experiment

Subsamples of ca. 200 diapausing eggs from each sample were individually placed in 96-multiwell plates (NUNC) and induced to hatch under constant light, 6 g L^−1^ salinity and 25 °C (standard hatching conditions^64^). These well plates were monitored daily for 28 days in order to estimate the diapausing egg hatching fraction and the timing of hatching.

### RNA extraction

The remaining 5000 diapausing eggs of each sample were subjected to two consecutive washes with DNase/RNase-free water (Gibco, Life Technologies). Once washed, the diapausing eggs of each sample were spun down in 1.5-mL microcentrifuge tubes and any remaining water was removed. Total RNA of each sample was isolated using the TRIzol Plus RNA Purification kit (Ambion, Life Technologies, USA) following the manufacturer’s instructions. In the first step of RNA isolation, once TRIzol reagent was added, a pellet pestle motor was used to homogenize the sample and assess the lysis of all diapausing eggs. DNase treatment during RNA purification was performed to reduce the amount of genomic DNA. At the end of the RNA extraction protocol each sample was quantified and the ratios 260/280 and 260/230 were assessed with a NanoDrop spectrophotometer (Thermo Scientific). Since the NFD condition was started one month earlier than the FD condition, the total RNA isolated from NFD samples was kept at − 80 °C. When the 12 samples of RNA were obtained, they were sent frozen to the *Servei Central de Suport a la Investigació Experimental* (SCSIE) of the University of Valencia (Spain) where RNA-Seq was performed.

### RNA-Seq

In the Genomic core facility at SCSIE the samples were quantified using a Qubit 2.0 Fluorometer (Life Technologies) and the purity and integrity of the RNA was assessed using electrophoresis in a Bioanalyzer 2100 (Agilent). After this quality control, cDNA libraries were constructed using TruSeq stranded mRNA (Illumina) with an enrichment of mRNA using the standard poly-A based method followed by chemical fragmentation and cDNA synthesis. Libraries were sequenced using 75 nt single-read sequencing on an Illumina NextSeq 500 platform. The quality of the raw reads was assessed using FastQC version 0.11.5 (www.bioinformatics.babraham.ac.uk/projects/fastqc/). Adapter sequences were trimmed and reads < 20 nt were discarded.

### Data analysis

#### Hatching data

The fraction of hatched eggs for each laboratory population and diapause condition assayed (NFD and FD) was estimated excluding deteriorated eggs^64^. The pure effects and interaction of the fixed-effect factors “regime” and “diapause condition” on the hatching fraction of diapausing eggs were analysed using a generalized linear mixed-effect model (GLMM) for a split-plot design considering binomial errors and logit as link function^81^. Factor “population”, nested within “regime”, was treated as a random effect whose significance was tested by means of a likelihood ratio test (LRT) comparing a model with all effects versus a model in which the “population” effect was not included. The timing of hatching was assessed by means of Kaplan–Meyer survival analysis of the cumulative hatching curves of the different populations, and the median hatching (H_50_) times (time in days needed for 50% of eggs to hatch) were estimated by linear interpolation. Censoring was applied to diapausing eggs that did not hatch by the end of the experiment. Cox’s proportional hazard model^82^ was used to test if there were differences in the timing of hatching among regimes and diapause conditions. Synchrony of diapausing egg hatching was estimated for each laboratory population by calculating the interquartile range (IQR) as a measure of dispersion. Thus, the higher the IQR, the greater the hatching asynchrony. Differences between selective regimes (P vs U) and between diapause conditions (FD vs NFD) in the IQR were assessed using a linear mixed-effect model (LMM). All analyses were performed in R, version 3.2.2^83^. GLMM and LRT were implemented by means of the *glmer* and *anova* functions of the “lme4” package^84^. LMM was performed using the *lmer* function of the same package. For Kaplan-Meyer survival analysis and Cox’s model fitting, the *Surv* and *coxph* functions from the “survival” package^85^ were used.

#### Transcriptome assembly

Transcriptome assembly was performed using the Tuxedo protocol^86^. RNA-Seq reads were mapped to a draft genome of *B. plicatilis* s.s.^87^ via* TopHat* v. 2.1.1^88^ using the following parameters: -I/-max-intron-length = 15,000; -i/-min-intron-length = 10; -max-multihits = 1; -read-gap-length = 1; -read-realign-edit-dist = 0; -library-type fr-firststrand. For the rest of parameters default values were used. To assemble the aligned reads into transcripts, *Cufflinks* software^86^ was used with default parameters modified as in *TopHat’*s mapping*.* Maximum and minimum intron length in *TopHat* and *Cufflinks* was assessed using the reference genome of *B. plicatilis* s.s. The values applied in the abovementioned parameters intended to follow a conservative approach to detect differential gene expression. Finally, *RSEM* (RNA-Seq by Expectation Maximization^89^) was used for quantifying both gene and isoform abundances based on the read mappings. Although the abundance of gene transcripts is typically expressed as RPKM^88^, the raw counts of reads mapped to transcripts were also used in subsequent analyses (see below).

#### Differential expression of genes

Expression data for genes in the dataset were fitted to the split-plot design described above (Fig. 5) using the “variancePartition” package^90^ in R, which allowed for the handling of fixed (“regime” and “diapause condition”) and random effect (“population”) factors. The *dream* function within this package was used to test for differential expression associated with each factor. Prior to model fitting, raw counts of reads were transformed to log_2_-counts per million mapped reads using the *voom* function in the “limma” package^91^]. We considered a gene to be expressed if it had at least five reads, and we only chose for analysis the genes expressed in at least four samples.

Differentially expressed genes (DEGs) associated with the “regime” and “diapause condition” factors were assessed separately in two alternative ways. On one hand, DEGs were ordered as usual by taking into account the log_2_-fold change (log_2_-FC) values between the levels of each factor and their associated *p*-values. To estimate the proportion of true null hypotheses among the whole set of statistical tests performed we used the least slope method^92^ implemented in the *estim.p0* function of package “cp4p” in R^93^. On the other hand, DEGs were also ordered by the proportion of expression variance explained by each factor. The latter was the preferred criterion for ordination in our analyses since genes with low total variability in expression may still be highly associated with a given factor even if the fold change between the levels of that factor is low. Notwithstanding, the differential expression data based on log_2_-FC and associated *p*-values were still used to validate the results of the variation partitioning approach.

#### Comparison of among-replicate dispersion of gene expression levels between selective regimes

We used the “DESeq2” package^94^ in R to estimate the dispersion of gene expression levels among biological replicates separately for samples of the two selective regimes. Gene-wise dispersion values are expected to reflect biological variation in true proportions of transcripts among replicates. They are estimated from few replicates by assuming a similar mean–variance relationship among genes^95^. In addition, we computed dissimilarities of gene expression profiles among samples using Poisson distance^96^. We tested for homogeneity of dispersions between the predictable and unpredictable regimes using the *betadisper* function of the “vegan*”* package^97^, that is, whether samples from the unpredictable regime were more different in terms of gene expression profiles within regime than samples from the predictable regime.

#### Functionality assignment of DEGs and Gene Ontology (GO)

The *gff2fasta* tool of CGAT toolkit^98^ was used to extract the sequences of the DEGs. Then sequentially, we used TransDecoder (https://github.com/TransDecoder/TransDecoder/wiki) to identify the proteins encoded within each transcript, and InterProScan^99^ to assign functional annotations to the proteins identified by TransDecoder.

We used the R package “TopGO”^100^ to run a gene ontology (GO) enrichment analysis among the genes that were differentially expressed between selective regimes or hatching conditions using a Kolmogorov–Smirnov test and a combination of three algorithms (*weight01*, *lea* and *elim*). GO terms that were significantly associated with the factor in question according to all three algorithms were chosen for further analysis. Because the association of expression with each classification factor was quantified in two different ways (see “Differentially expressed genes” section), the functional enrichment analysis was run separately on the dataset of DEGs ordered by the proportion of expression variance explained by the factor concerned, and the dataset ordered by the *p*-values of the corresponding tests of differential expression. Both approaches were compared to validate and generalize the results of differential gene expression associated with the study factors.

Finally, we used information from previous studies that have provided very useful genetic resources for the discovery and function assignment of genes associated with diapause and embryonic development in *B. plicatilis*^34–36^, *Brachionus calyciflorus*^101^, and *Brachionus manjavacas*^58^ as well as in other aquatic and terrestrial invertebrates. This information was used to (1) perform a tentative assignment of GO terms to diapause maintenance and termination, and (2) establish links between the observed hatching phenotypes and their corresponding RNA expression profiles.

## Supplementary information

Supplementary Information.

## Data Availability

The sequence data generated during the current study are available in compressed fastq format in the Zenodo repository (https://zenodo.org/record/3953665#.Xx63nqYp5hE). The whole analysis described here is registered and documented in executable scripts available in the GitHub repository https://github.com/ignasilucas/Brachionus.

## References

[1] García-Roger EM, Carmona MJ, Serra M (2019). Facing adversity: Dormant embryos in rotifers. Biol. Bull..

[2] Denlinger DL (2002). Regulation of diapause. Annu. Rev. Entomol..

[3] Reynolds JA, Hand SC (2009). Embryonic diapause highlighted by differential expression of mRNAs for ecdysteroidogenesis, transcription and lipid sparing in the cricket *Allonemobius socius*. J. Exp. Biol..

[4] Ricci C (2001). Dormancy patterns in rotifers. Hydrobiologia.

[5] Poelchau MF, Reynolds JA, Elsik CG, Denlinger DL, Armbruster PA (2013). Deep sequencing reveals complex mechanisms of diapause preparation in the invasive mosquito, *Aedes albopictus*. Proc. R. Soc. B..

[6] Alekseev VR, De Stasio BT, Gilbert JJ, Ravera O, Alekseev VR, De Stasio BT, Gilbert JJ (2007). Preface. Diapause in Aquatic Invertebrates, Theory and Human Use.

[7] Hand SC, Podrabsky JE (2000). Bioenergetics of diapause and quiescence in aquatic animals. Thermochim. Acta.

[8] Ślusarczyk M, Chlebicki W, Pijanowska J, Radzikowski J (2019). The role of the refractory period in diapause length determination in a freshwater crustacean. Sci. Rep..

[9] Tauber MJ, Tauber CA, Masaki S (1986). Seasonal Adaptations of Insects.

[10] Alekseev VR, De Stasio BT, Gilbert JJ (2012). Diapause in Aquatic Invertebrates, Theory and Human Use.

[11] García-Roger EM, Carmona MJ, Serra M (2017). Modes, mechanisms and evidence of bet hedging in rotifer diapause traits. Hydrobiologia.

[12] Cohen D (1966). Optimizing reproduction in a randomly varying environment. J. Theor. Biol..

[13] Seger J, Brockmann HJ, Harvey PH, Partridge L (1987). What is bet-hedging?. Oxford Surveys in Evolutionary Biology.

[14] Philippi T, Seger J (1989). Hedging one's evolutionary bets, revisited. Trends Ecol. Evol..

[15] Simons AM (2011). Modes of response to environmental change and the elusive empirical evidence for bet hedging. Proc. R. Soc. B Biol. Sci..

[16] Menu F, Desouhant E (2002). Bet-hedging for variability in life cycle duration: bigger and later-emerging chestnut weevils have increased probability of a prolonged diapause. Oecologia.

[17] Franch-Gras L, García-Roger EM, Serra M, Carmona MJ (2017). Adaptation in response to environmental unpredictability. Proc. R. Soc. B Biol. Sci..

[18] Tarazona E, García-Roger EM, Carmona MJ (2017). Experimental evolution of bet hedging in rotifer diapause traits as a response to environmental unpredictability. Oikos.

[19] Koštál V (2006). Eco-physiological phases of insect diapause. J. Insect Physiol..

[20] Tammariello SP, Denlinger DL (1998). G0/G1 cell cycle arrest in the brain of *Sarcophaga crassipalpis* during pupal diapause and the expression pattern of the cell cycle regulator, proliferating cell nuclear antigen. Insect. Biochem. Mol. Biol..

[21] Denekamp NY, Reinhardt R, Kube M, Lubzens E (2010). Late embryogenesis abundant (LEA) proteins in nondesiccated, encysted, and diapausing embryos of rotifers. Biol. Repr..

[22] Qiu Z, MacRae TH, Lubzens E, Cerdà BJ, Clark MS (2010). A molecular overview of diapause in embryos of the crustacean, *Artemia franciscana*. Dormancy and Resistance in Harsh Environments.

[23] Ziv T (2017). Dormancy in embryos: Insight from hydrated encysted embryos of an aquatic invertebrate. Mol. Cell. Proteomics.

[24] Roncalli V (2018). Physiological characterization of the emergence from diapause: A transcriptomics approach. Sci. Rep..

[25] Rozema E (2019). Metabolomics reveals novel insight on dormancy of aquatic invertebrate encysted embryos. Sci. Rep..

[26] Vanvlasselaer E, De Meester L, Lubzens E, Cerdà BJ, Clark MS (2010). An exploratory review on the molecular mechanisms of diapause termination in the waterflea. Daphnia in Dormancy and Resistance in Harsh Environments.

[27] Declerck SAJ, Papakostas S (2017). Monogonont rotifers as model systems for the study of micro-evolutionary adaptation and its eco-evolutionary implications. Hydrobiologia.

[28] Serra M, García-Roger EM, Ortells R, Carmona MJ (2019). Cyclically parthenogenetic rotifers and the theories of population and evolutionary ecology. Limnetica.

[29] García-Roger EM, Serra M, Carmona MJ (2014). Bet-hedging in diapausing egg hatching of temporary rotifer populations—A review of models and new insights. Int. Rev. Hydrobiol..

[30] Ricci C, Pagani M (1997). Desiccation of *Panagrolaimus rigidus* (Nematoda): Survival, reproduction and the influence on the internal clock. Hydrobiologia.

[31] Gordon G, Headrick DH (2001). A Dictionary of Entomology.

[32] Fan L, Lin J, Zhong Y, Liu J (2013). Shotgun proteomic analysis on the diapause and nondiapause eggs of domesticated silkworm *Bombyx mori*. PLoS ONE.

[33] Schröder T (2005). Diapause in monogonont rotifers. Hydrobiologia.

[34] Denekamp NY (2009). Discovering genes associated with dormancy in the monogonont rotifer *Brachionus plicatilis*. BMC Genomics.

[35] Denekamp NY (2011). The expression pattern of dormancy-associated genes in multiple life-history stages in the rotifer *Brachionus plicatilis*. Hydrobiologia.

[36] Clark MS (2012). Long-term survival of hydrated resting eggs from *Brachionus plicatilis*. PLoS ONE.

[37] Waterworth WM, Bray CM, West CE (2015). The importance of safeguarding genome integrity in germination and seed longevity. J. Exp. Bot..

[38] Sim C, Denlinger DL (2011). Catalase and superoxide dismutase-2 enhance survival and protect ovaries during overwintering diapause in the mosquito *Culex pipiens*. J. Insect Physiol..

[39] Ragland GJ, Denlinger DL, Hahn DA (2010). Mechanisms of suspended animation are revealed by transcript profiling of diapause in the flesh fly. Proc. Natl. Acad. Sci. USA.

[40] Duceppe MO (2017). Analysis of survival and hatching transcriptomes from potato cyst nematodes, *Globodera rostochiensis* and *G. pallida*. Sci. Rep..

[41] Wise MJ, Tunnacliffe A (2004). POPP the question: What do LEA proteins do?. Trends Plant. Sci..

[42] García-Roger EM, Ortells R (2018). Trade-offs in rotifer diapausing egg traits: Survival, hatching, and lipid content. Hydrobiologia.

[43] Hand SC, Menze MA, Toner M, Boswell L, Moore D (2011). LEA proteins during water stress: Not just for plants anymore. Annu. Rev. Physiol..

[44] Crowe JH (2001). The trehalose myth revisited: Introduction to a symposium on stabilization of cells in the dry state. Cryobiology.

[45] Moore DS, Hand SC (2016). Cryopreservation of lipid bilayers by LEA proteins from *Artemia franciscana* and trehalose. Cryobiology.

[46] Clegg JS (1965). Origin of trehalose and its significance during formation of encysted dormant embryos of *Artemia Salina*. Comp. Biochem. Physiol..

[47] Caprioli M (2004). Trehalose in desiccated rotifers: A comparison between a bdelloid and a monogonont species. Comp. Biochem. Physiol..

[48] Li T, Liu L, Zhang L, Liu N (2015). Role of G-protein-coupled receptor-related genes in insecticide resistance of the mosquito, *Culex quinquefasciatus*. Sci. Rep..

[49] Hommaa T (2006). G protein-coupled receptor for diapause hormone, an inducer of *Bombyx* embryonic diapause. Biochem. Biophys. Res. Comm..

[50] Jones SJ (2001). Changes in gene expression associated with developmental arrest and longevity in *Caenorhabditis elegans*. Genome Res..

[51] Fielenbach N, Antebi A (2008). *C. elegans* dauer formation and the molecular basis of plasticity. Genes Dev..

[52] Hand SC, Denlinger DL, Podrabsky JE, Roy R (2016). Mechanisms of animal diapause: recent developments from nematodes, crustaceans, insects, and fish. Am. J. Physiol. Regul. Integr. Comp. Physiol..

[53] Woll SC, Podrabsky JE (2017). Insulin-like growth factor signaling regulates developmental trajectory associated with diapause in embryos of the annual killifish *Austrofundulus limnaeus*. J. Exp. Biol..

[54] Yu CT, Hirsh D (1967). The stimulatory effect of ammonium or potassium ions on the activity of leucyl-tRNA synthetase from *Escherichia coli*. Biochim. Biophys. Acta.

[55] Beck SD, Shane JL, Garland JA (1969). Ammonium-induced termination of diapause in the European corn borer, *Ostrinia nubilalis*. J. Insect. Physiol..

[56] Birnbaumer L (2007). Expansion of signal transduction by G proteins. The second 15 years or so: From 3 to 16 alpha subunits plus betagamma dimers. Biochim. Biophys. Acta.

[57] Dumont H, Casier P, Munuswamy N, De Wasche C (1992). Cyst hatching in Anostraca accelerated by retinoic acid, amplified by calcium ionosphore A23187, and inhibited by calcium-channel blockers. Hydrobiologia.

[58] Kim HJ (2015). Light-dependent transcriptional events during resting egg hatching of the rotifer *Brachionus manjavacas*. Mar. Genomics.

[59] Boschetti C, Ricci C, Sotgia C, Fascio U (2005). The development of a bdelloid egg: A contribution after 100 years. Hydrobiologia.

[60] Bonneau B, Popgeorgiev N, Prudent J, Gillet G (2011). Cytoskeleton dynamics in early zebrafish development. A matter of phosphorylation?. Bioarchitecture.

[61] Eno C, Solanki B, Pelegri F (2016). Aura (mid1ip1l) regulates the cytoskeleton at the zebrafish egg-to-embryo transition. Development.

[62] Cáceres CE, Schwalbach MS (2001). How well do laboratory experiments explain field patterns of zooplankton emergence?. Freshw. Biol..

[63] De Stasio BT (2004). Diapause in calanoid copepods: Within-clutch hatching patterns. J. Limnol..

[64] García-Roger EM, Carmona MJ, Serra M (2006). Patterns in rotifer diapausing egg banks: Density and viability. J. Exp. Mar. Biol. Ecol..

[65] Helland S, Nejstgaard C, Fyhn JJ, Egge JK, Båmstedt U (2003). Effects of starvation, season, and diet on the free amino acid and protein content of *Calanus finmarchicus* females. Mar. Biol..

[66] Skottene E (2019). The β-oxidation pathway is downregulated during diapause termination in *Calanus* copepods. Sci. Rep..

[67] Tan Q, Liu W, Zhu F, Lei C, Wang X (2016). Fatty acid synthase 2 contributes to diapause preparation in a beetle by regulating lipid accumulation and stress tolerance genes expression. Sci. Rep..

[68] Gilbert JJ, Schröder T (2004). Rotifers from diapausing, fertilized eggs: Unique features and emergence. Limnol. Oceanogr..

[69] Alekseev VR, Hwang J-S, Tseng M-H (2006). Diapause in aquatic invertebrates: What's known and what's next in research and medical application?. J. Mar. Sci. Tech..

[70] Gilbert JJ, Alekseev VR, De Stasio BT, Gilbert JJ (2012). Timing of diapause in monogonont rotifers. Mechanisms and Strategies in Diapause in Aquatic Invertebrates. Theory and Human Use.

[71] Koštál V, Štětina T, Poupardin R, Korbelová J, Bruce AW (2017). Conceptual framework of the eco-physiological phases of insect diapause development justified by transcriptomic profiling. Proc. Natl. Acad. Sci. USA.

[72] Podrabsky JE, Hand SC (2015). Physiological strategies during animal diapause: Lessons from brine shrimp and annual killifish. J. Exp. Biol..

[73] Zahradka K (2006). Reassembly of shattered chromosomes in *Deinococcus radiodurans*. Nature.

[74] Gladyshev E, Meselson M (2008). Extreme resistance of bdelloid rotifers to ionizing radiation. Proc. Natl. Acad. Sci. USA.

[75] Kim RO (2011). Ultraviolet B retards growth, induces oxidative stress, and modulates DNA repair-related gene and heat shock protein gene expression in the monogonont rotifer, *Brachionus* sp. Aquat. Toxicol..

[76] Han J (2014). Sublethal gamma irradiation affects reproductive impairment and elevates antioxidant enzyme and DNA repair activities in the monogonont rotifer *Brachionus koreanus*. Aquat Toxicol..

[77] Hagiwara A, Hoshi N, Kawahara F, Tominaga K, Hirayama K (1995). Resting eggs of the marine rotifer *Brachionus plicatilis* Müller: Development and effect of irradiation on hatching. Hydrobiologia.

[78] IPCC (2013). Climate Change 2013: The Physical Science Basis. Contribution of Working Group I to the Fifth Assessment Report of the Intergovernmental Panel on Climate Change.

[79] Pourriot R, Snell TW (1983). Resting eggs of rotifers. Hydrobiologia.

[80] Altman N, Krzywinski M (2015). Split plot design. Nat. Meth..

[81] Nelder JA, Wedderburn RWM (1972). Generalized linear models. J. Roy. Stat. Soc. Ser. A.

[82] Cox DR (1972). Regression models and life-tables (with discussion). J. R. Statist. Soc. B.

[83] R Development Core Team. *R: A language and environment for statistical computing*. R Foundation for Statistical Computing, Vienna. https://www.R-project.org/ (2017).

[84] Bates D, Mächler M, Bolker B, Walker S (2015). Fitting linear mixed-effects models using lme4. J. Stat. Softw..

[85] Therneau TM, Grambsch PM (2020). Modeling Survival Data: Extending the Cox Model.

[86] Trapnell C (2012). Differential gene and transcript expression analysis of RNA-seq experiments with TopHat and Cufflinks. Nat. Protoc..

[87] Franch-Gras L (2018). Genomic signatures of local adaptation to the degree of environmental unpredictability in rotifers. Sci. Rep..

[88] Trapnell C (2010). Transcript assembly and quantification by RNA-Seq reveals unannotated transcripts and isoform switching during cell differentiation. Nat. Biotech..

[89] Li B, Dewey CN (2011). RSEM: Accurate transcript quantification from RNA-Seq data with or without a reference genome. BMC Bioinformatics.

[90] Hoffman GE, Schadt EE (2016). VariancePartition: Interpreting drivers of variation in complex gene expression studies. BMC Bioinformatics.

[91] Ritchie ME (2015). Limma powers differential expression analyses for RNA-sequencing and microarray studies. Nucleic Acids Res..

[92] Benjamini Y, Hochberg Y (2000). On the adaptive control of the false discovery rate in multiple testing with independent statistics. J. Educ. Behav. Stat..

[93] Gianetto GQ (2016). Calibration plot for proteomics: A graphical tool to visually check the assumptions underlying FDR control in quantitative experiments. Proteomics.

[94] Love MI, Huber W, Anders S (2014). Moderated estimation of fold change and dispersion for RNA-seq data with DESeq2. Genome Biol..

[95] McCarthy DJ, Chen Y, Smyth GK (2012). Differential expression analysis of multifactor RNA-Seq experiments with respect to biological variation. Nuc. Acids Res..

[96] Witten D (2011). Classification and clustering of sequencing data using a Poisson model. Ann. Appl. Stat..

[97] Anderson MJ (2006). Distance-based tests for homogeneity of multivariate dispersions. Biometrics.

[98] Sims D (2014). CGAT: Computational genomics analysis toolkit. Bioinformatics.

[99] Jones P (2014). InterProScan 5: Genome-scale protein function classification. Bioinformatics.

[100] Alexa A, Rahnenführer J (2020). TopGO: Enrichment analysis for gene ontology. R package version 2.40.0. Bioconductor.

[101] Hanson SJ, Stelzer C-P, Welch DB, Logsdon J (2013). Comparative transcriptome analysis of obligately asexual and cyclically sexual rotifers reveals genes with putative functions in sexual reproduction, dormancy, and asexual egg production. BMC Genomics.

