# Supplementary material for: Gene expression in diapausing rotifer eggs in response to divergent environmental predictability regimes
Source: Sci Rep. 2020 Dec 7;10:21366. doi: 10.1038/s41598-020-77727-2 (PMC7721884; doi:10.1038/s41598-020-77727-2)
Supplement: Supplementary file 1 — Supplementary Information. [file 41598_2020_77727_MOESM1_ESM.pdf]

Supplementary Material

Gene expression in diapausing rotifer eggs in response to divergent environmental predictability regimes

Eva Tarazona[1§], J. Ignacio Lucas-Lledó[1§], María José Carmona[1] & Eduardo M. García-Roger[1]*

[1] Institut Cavanilles de Biodiversitat i Biologia Evolutiva, Universitat de València, Valencia, Spain.

* Corresponding author: eduardo.garcia@uv.es

[§] Both authors contributed equally to this work.

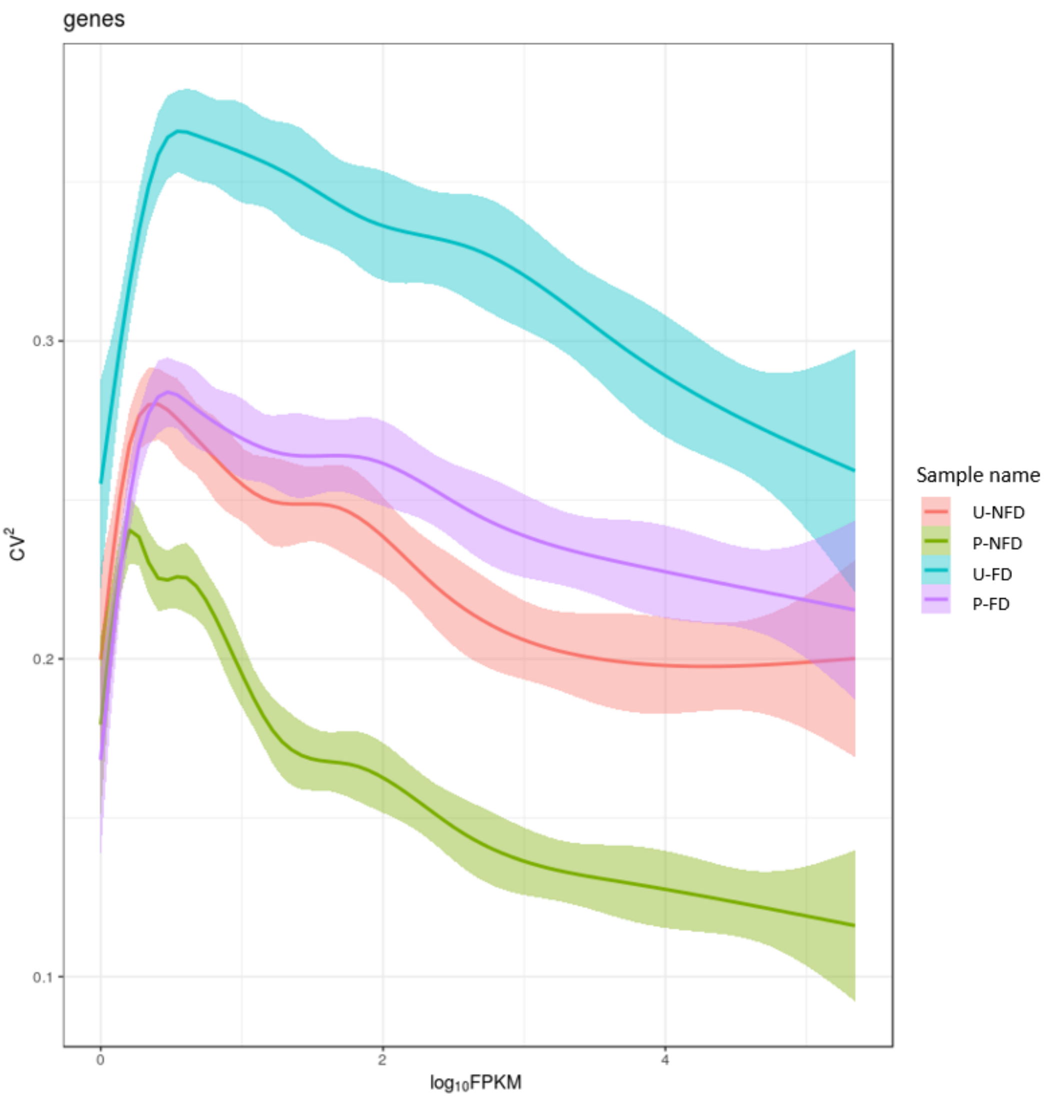

Figure S1. RPKM density distribution plot (the squared coefficient of variation (CV^2) vs log_{10}FPKM) per selective regime and diapause condition.

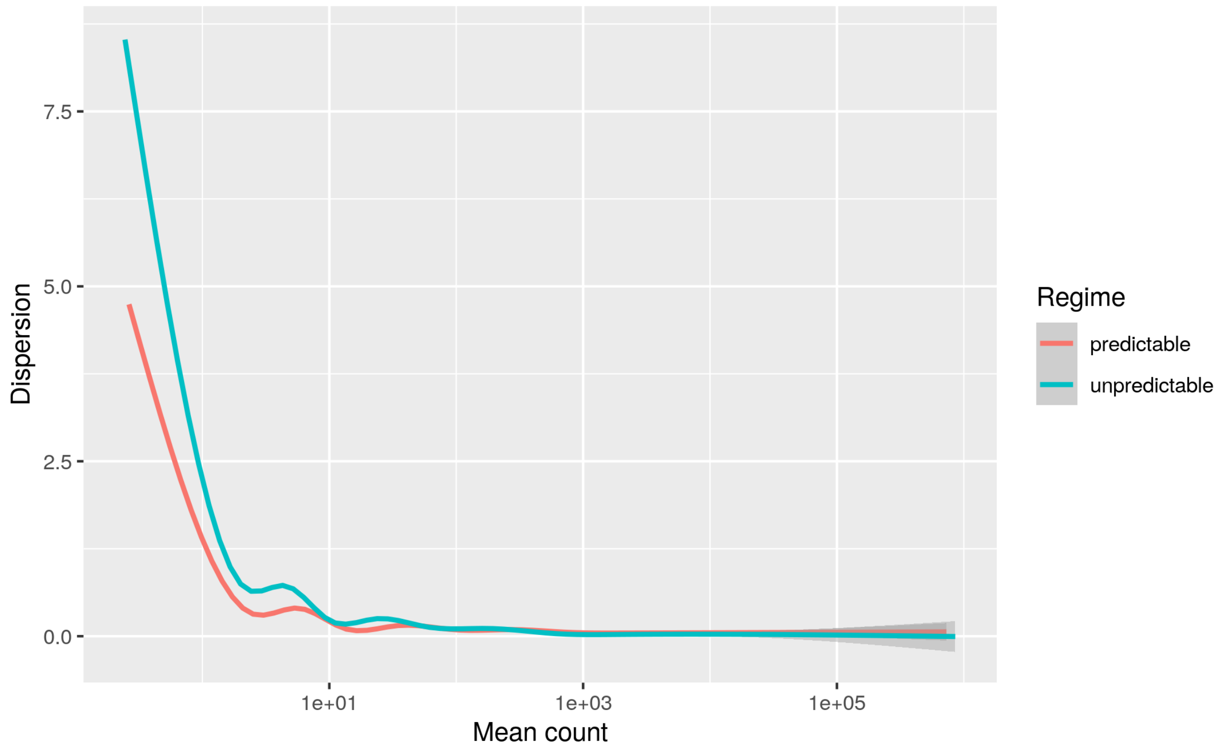

Figure S2. Estimated relationship between biological variation in gene expression between replicates (dispersion) and average level of expression among replicates, measured by a normalized number of counts. Estimates of gene-wise dispersions were obtained using the *estimateDispersion()* function of the *DESeq2* package, separately for samples from the two selective regimes.

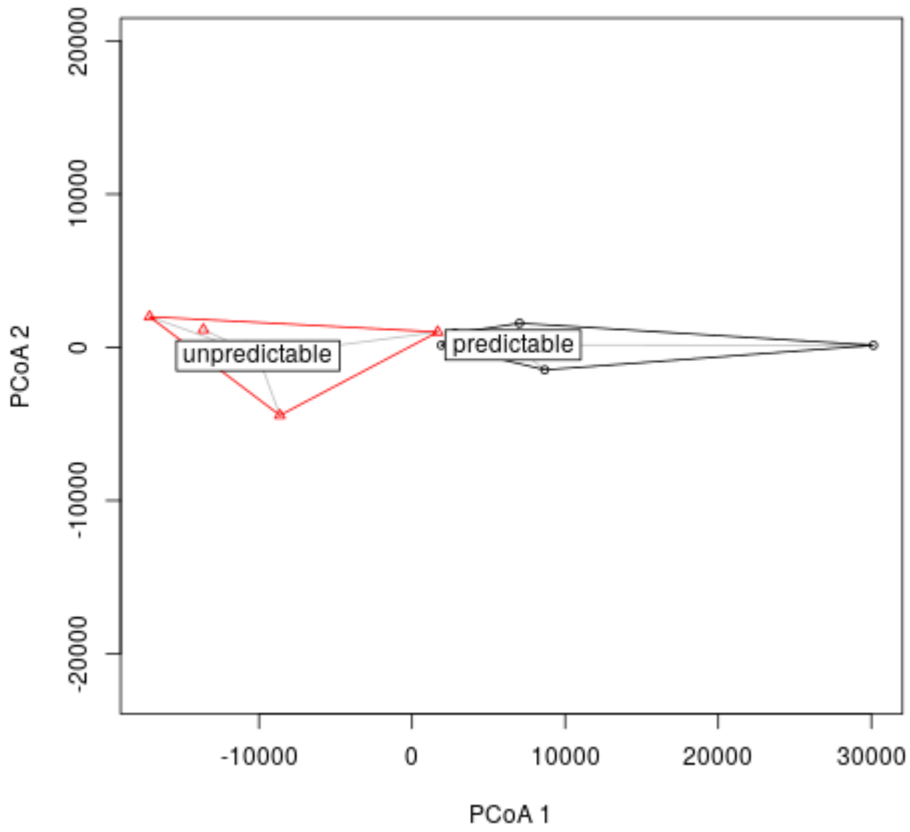

Figure S3. Principal coordinates of gene expression profiles based on Poisson distances. Distances of samples to their regime-specific centroid are not significantly different between selective regimes (*p*-value = 0.883).

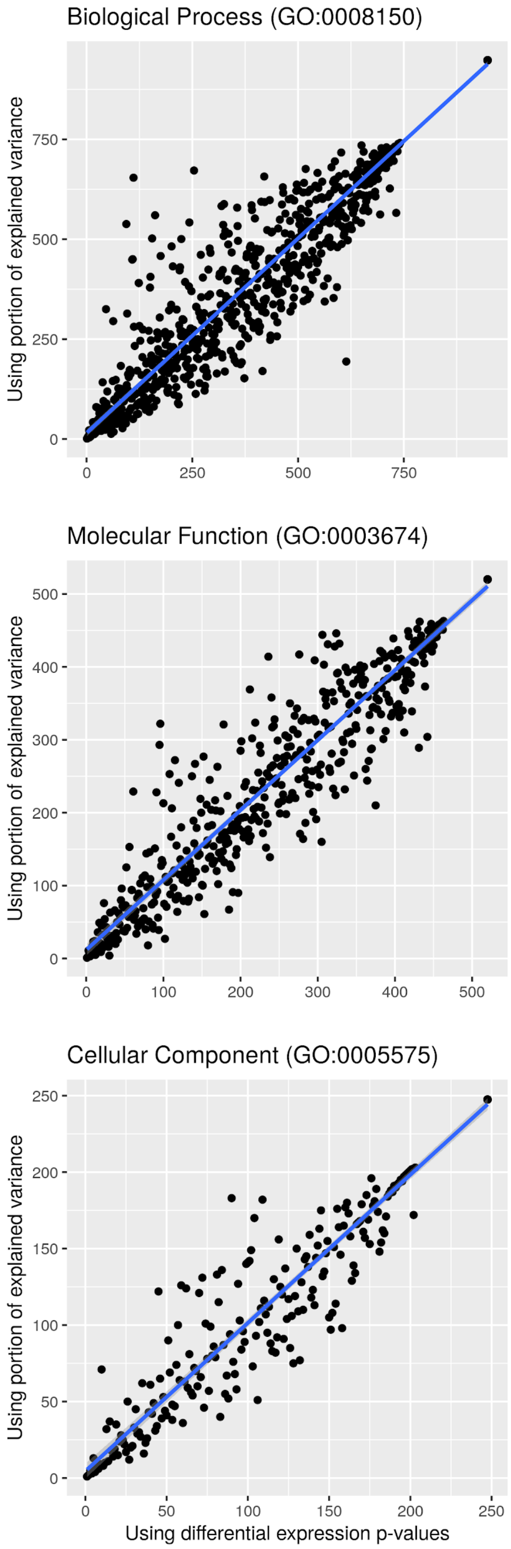

Figure S4. Ordination of gene ontology (GO) terms according to their significance in the enrichment analyses based on either the differential expression *p*-values or the portion of variance explained by regime.

Table S1. Gene Ontology (GO) specific terms for biological process (GO:0008150) significantly associated with selective regime according to all three algorithms used in enrichment analysis. *p*-values are derived from Kolmogorov-Smirnov tests.

GO ID	GO Term	*p*-value
GO:0055085	transmembrane transport	< 0.001
GO:0006508	proteolysis	< 0.001
GO:0055114	oxidation-reduction process	< 0.001
GO:0007186	G protein-coupled receptor signalling pathway	< 0.001
GO:0006468	protein phosphorylation	< 0.001
GO:0006813	potassium ion transport	< 0.001
GO:0006355	regulation of transcription, DNA-templated	< 0.001
GO:0006289	nucleotide-excision repair	< 0.001
GO:0034220	ion transmembrane transport	0.001
GO:0006470	protein dephosphorylation	0.001
GO:0006812	cation transport	0.002
GO:0003341	cilium movement	0.002
GO:0060271	cilium assembly	0.002
GO:0005992	trehalose biosynthetic process	0.003
GO:0007018	microtubule-based movement	0.003
GO:0006367	transcription initiation from RNA polymerase II promoter	0.004
GO:0046039	GTP metabolic process	0.004
GO:0046129	purine ribonucleoside biosynthetic process	0.004
GO:0007160	cell-matrix adhesion	0.005
GO:0070836	caveola assembly	0.006
GO:0006821	chloride transport	0.007
GO:0006979	response to oxidative stress	0.007
GO:0043161	proteasome-mediated ubiquitin-dependent protein catabolic process	0.008
GO:0051260	protein homooligomerization	0.008
GO:0008272	sulphate transport	0.009

Table S2. Gene ontology (GO) specific terms for molecular function (GO:0003674) significantly associated with selective regime according to all three algorithms used in enrichment analysis. *p*-values are derived from Kolmogorov-Smirnov tests.

GO ID	GO Term	*p*-value
GO:0005515	protein binding	< 0.001
GO:0005509	calcium ion binding	< 0.001
GO:0003774	motor activity	< 0.001
GO:0004930	G protein-coupled receptor activity	< 0.001
GO:0004252	serine-type endopeptidase activity	< 0.001
GO:0005524	ATP binding	< 0.001
GO:0004672	protein kinase activity	< 0.001
GO:0022857	transmembrane transporter activity	< 0.001
GO:0042626	ATPase activity, coupled to transmembrane movement of substances	0.001
GO:0004198	calcium-dependent cysteine-type endopeptidase activity	0.001
GO:0005525	GTP binding	0.001
GO:0061630	ubiquitin protein ligase activity	0.001
GO:0004181	metallocarboxypeptidase activity	0.001
GO:0005230	extracellular ligand-gated ion channel activity	0.002
GO:0005249	voltage-gated potassium channel activity	0.002
GO:0005507	copper ion binding	0.003
GO:0051015	actin filament binding	0.003
GO:0016840	carbon-nitrogen lyase activity	0.004
GO:0004550	nucleoside diphosphate kinase activity	0.005
GO:0004601	peroxidase activity	0.005
GO:0030246	carbohydrate binding	0.005
GO:0005200	structural constituent of cytoskeleton	0.006
GO:0016491	oxidoreductase activity	0.007
GO:0003777	microtubule motor activity	0.008
GO:0047631	ADP-ribose diphosphatase activity	0.008

Table S3. Gene ontology (GO) specific terms for cellular component (GO:0005575) significantly associated with selective regime according to all three algorithms used in enrichment analysis. *p*-values are derived from Kolmogorov-Smirnov tests.

GO ID	GO Term	*p*-value
GO:0016020	membrane	< 0.001
GO:0016459	myosin complex	< 0.001
GO:0008076	voltage-gated potassium channel complex	< 0.001
GO:0005887	integral component of plasma membrane	0.001
GO:0005576	extracellular region	0.001
GO:0090575	RNA polymerase II transcription factor complex	0.001
GO:0005634	nucleus	0.003
GO:0005886	plasma membrane	0.003
GO:0005743	mitochondrial inner membrane	0.004
GO:0005929	cilium	0.004
GO:0005741	mitochondrial outer membrane	0.009
